# How Does Porcine Epidemic Diarrhea Virus Escape Host Innate Immunity?

**DOI:** 10.3390/pathogens14100971

**Published:** 2025-09-25

**Authors:** Jinyuan Li, Hao Lu, Gaowei Hu, Shengmei Pang, Yuqing Xie, Guoqiang Zhu, Xueyan Ding

**Affiliations:** 1 College of Veterinary Medicine, Henan Agricultural University, Zhengzhou 450046, China; 2 Molecular Biology Laboratory, Zhengzhou Normal University, Zhengzhou 450044, China; 3 College of Life Sciences, Taizhou University, Taizhou 318000, China; 4 College of Veterinary Medicine, Yangzhou University, Yangzhou 225009, China; 5 Henan Province Key Laboratory of Animal Food Pathogens Surveillance, Zhengzhou 450046, China; 6 Ministry of Education Key Laboratory for Animal Pathogens and Biosafety, Zhengzhou 450046, China

**Keywords:** PEDV, PED, innate immunity, immune escape, control strategies and therapeutics

## Abstract

Porcine epidemic diarrhea virus (PEDV), the causative agent of porcine epidemic diarrhea (PED), induces vomiting, watery diarrhea, and severe dehydration in pigs. It exhibits particularly high lethality in neonatal piglets, posing a significant threat to the global swine industry and inflicting substantial economic losses. The host innate immune system serves as the primary defense against viral invasion; however, PEDV employs multiple strategies to evade this response. This review systematically summarizes the multiple molecular mechanisms by which PEDV evaded the host’s innate immunity, including interfering with host intracellular signaling pathways by virally encoded proteins, antagonizing the host’s antiviral factors and related immune genes to suppress the innate immune responses, and regulating the autophagy process of the host cells, thereby achieving the escape of the host’s innate immunity. A comprehensive understanding of how PEDV subverts innate immunity is crucial for developing effective control strategies and therapeutics. This review aims to provide novel insights and potential targets for combating PED.

## 1. Introduction

Porcine epidemic diarrhea (PED) is an acute enteric disease primarily caused by porcine epidemic diarrhea virus (PEDV), characterized by diarrhea, vomiting, reduced feed intake, and dehydration. While pigs of all ages and breeds are susceptible, the virus poses the most severe threat to piglets within the first 14 days of life, significantly endangering the swine industry and causing substantial global economic losses [1]. PEDV, classified within the genus Alphacoronavirus, is an enveloped, single-stranded positive-sense RNA virus [2]. Its approximately 28-kb genome comprises a 5′ untranslated region (UTR), at least seven open reading frames (ORF1a, ORF1b, and ORF2 to ORF6), and a 3′ UTR. ORF1a and ORF1b represent the primary non-structural protein (NSP) coding regions. A 46-nt overlap between these ORFs facilitates ribosomal frameshifting, enabling translation of a large polyprotein (pp1ab) [2,3]. Subsequent proteolytic cleavage of pp1ab generates 16 NSPs (NSP1 to NSP16). The remaining ORFs near the 3′ terminus (ORF2 to ORF6) encode four structural proteins: the spike (S), envelope (E), membrane (M), and nucleocapsid (N) proteins.

Hosts have evolved a sophisticated immune system comprising innate and adaptive branches to counter pathogenic threats [4]. The innate immune system serves as the frontline defense through physicochemical barriers—including skin, mucosal surfaces, and chemical agents like enzymes and complement proteins—that block microbial invasion. Upon pathogen breach, infected cells release cytokines and chemokines to initiate inflammation, recruiting phagocytes and antigen-presenting cells (APCs) for pathogen clearance. For pathogens evading these mechanisms, the adaptive immune system activates via APC-mediated antigen presentation through MHC molecules, triggering pathogen-specific T and B lymphocyte responses that generate neutralizing antibodies and establish immunological memory. These first two tiers provide broad-spectrum, rapid protection primarily mediated by innate immunity during early infection [5]. As obligate intracellular pathogens, viruses are detected by host pattern recognition receptors (PRRs) that identify pathogen-associated molecular patterns (PAMPs) such as viral nucleic acids, inducing interferon (IFN) and pro-inflammatory cytokine production to establish an antiviral state [6]. However, viruses including PEDV have evolved counterstrategies to disrupt innate immune signaling, thereby facilitating viral replication and pathogenesis [7].

This review systematically elaborates on the multiple molecular mechanisms by which PEDV evades the host’s innate immunity. PEDV employs a sophisticated immune evasion strategy through coordinated actions of its structural, non-structural, and accessory proteins, which collectively undermine host innate immunity by disrupting intracellular signaling pathways, antagonizing antiviral effectors and immune-related genes, and hijacking cellular autophagy processes. It is noteworthy that the capacity of PEDV to evade host innate immunity constitutes the primary reason for its persistence in hosts and high transmission efficiency among swine populations. Consequently, elucidating the core mechanisms underlying this immune evasion is pivotal to overcoming the current bottleneck in PED control strategies. These mechanistically distinct yet functionally convergent interventions allow PEDV to systematically bypass innate immune surveillance and establish a proviral cellular environment. The elucidation of these molecular tactics not only advances our understanding of coronavirus pathogenesis but also provides a rational framework for developing targeted countermeasures, including protein-specific inhibitors, autophagy-modulating compounds, and structure-based vaccine designs with enhanced efficacy against this economically devastating swine pathogen.

## 2. Role of Virus-Encoded Proteins in the Escape of PEDV from Host Innate Immunity

### 2.1. Role of Structural Proteins in the Escape of PEDV from Host Innate Immunity

#### 2.1.1. S Protein

The PEDV S glycoprotein, as the largest structural protein on the viral envelope, orchestrates viral entry through a sophisticated two-step mechanism. This trimeric glycoprotein consists of S1 (residues 1–789) and S2 (790–1383) subunits, with the S1 domain harboring critical neutralizing epitopes (including S1^0^, S1^A^, COE, SS2, SS6, and C-terminal epitopes) and two functionally distinct receptor-binding domains (S1-NTD and S1-CTD) that collectively determine viral antigenicity and host cell attachment [8,9]. Following receptor engagement by S1, conformational changes expose proteolytic cleavage sites, leading to S1/S2 subunit dissociation and subsequent S2-mediated membrane fusion-a process initiated by fusion peptide insertion into host membranes [10,11]. The strategic distribution of immunodominant epitopes across the S protein, particularly within the S1 domain (aa 1–219, 435–485, 499–638, 748–755, 764–771) and C-terminus (aa 1368–1374), establishes this structural protein as a prime target for epitope-based vaccine design [12,13,14,15,16,17,18,19]. These molecular features not only elucidate PEDV’s invasion strategy but also provide a structural framework for developing targeted interventions against this economically significant swine pathogen [20].

**Figure 1:**
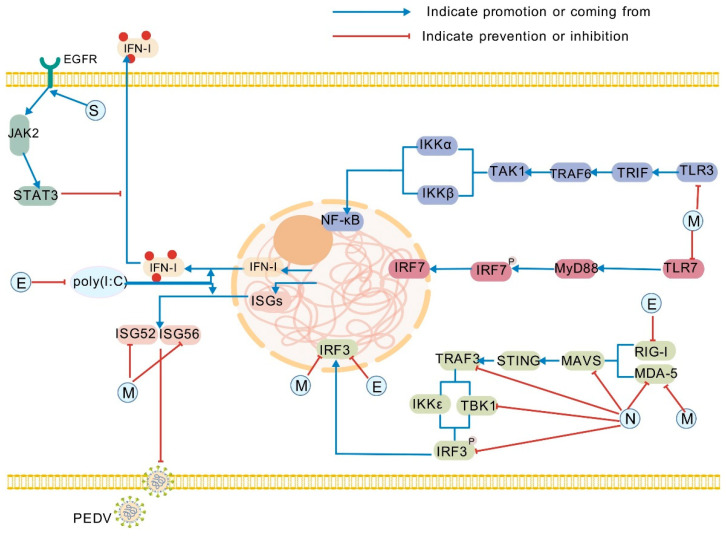
Role of structural proteins in the escape of PEDV from host innate immunity. Specifically, these structural proteins inhibit key nodes such as JAK2/STAT3, NF-κB, and IRF, interfere with PRRs such as TLR3, TLR7, and RIG-I/MDA-5, and antagonize IFN-stimulated factors. They can not only produce an antagonistic effect on the host innate immune response, but also assist the virus in evading the host innate immune defense through this antagonistic effect. EGFR: epidermal growth factor receptor; poly(I:C): polyinosinic-polycytidylic acid; JAK2: Janus kinase 2; STAT3: signal transducer and activator of transcription 3; TLR3: Toll-like receptor 3; MAVS: mitochondrial antiviral signaling protein; IKKα: IκB kinase alpha; IKKε: inhibitor of nuclear factor-κb epsilon kinase; STING: stimulator of interferon genes. This figure was created by BioGDP.com [23].

Type I interferons (IFN-I) serve as pivotal innate immune cytokines that trigger host antiviral defenses. Mechanistically, the S protein of PEDV directly engages the host epidermal growth factor receptor (EGFR). Subsequent EGFR activation initiates downstream JAK2-STAT3 signaling, wherein STAT3 functions as a transcriptional repressor of IFN-I production (Figure 1). This cascade consequently suppresses IFN-I-mediated antiviral responses [21]. Notably, the S1 subunit emerges as a potent inducer of apoptosis during PEDV infection. By promoting programmed cell death, S1 facilitates viral egress and enables evasion of intracellular immune surveillance—a strategy that enhances viral dissemination while counteracting host defense mechanisms [22]. Concurrently, the S protein interacts with the EGFR, suppressing the production of mucosal antiviral cytokines—notably the key mucosal interferon IFN-λ—thereby further compromising mucosal immune surveillance function [21].

#### 2.1.2. M Protein

The PEDV M protein, a highly conserved 227-amino acid type III glycoprotein, plays multifaceted roles in virion assembly and budding while serving as a key immunogenic structural component. During infection, M protein localizes throughout the cytoplasm and induces S-phase cell cycle arrest in porcine intestinal epithelial cells (IECs) by downregulating cyclin A [24]. Mechanistically, M protein interacts with eukaryotic translation initiation factor 3L (eIF3L), suppressing this negative regulator of viral replication to enhance PEDV propagation [25]. Furthermore, M protein exhibits broad antagonistic effects against innate immune sensors including RIG-I, TLR3, TLR7, and interferon regulatory factor 3 (IRF3). It specifically targets IRF7—a master transcriptional regulator of IFN-I production—by binding the IRF7 interaction domain (ID). This interaction impedes TBK1/IKKε-mediated phosphorylation, thereby inhibiting IRF7 dimerization, nuclear translocation, and subsequent IFN-I induction [26]. Additionally, M protein suppresses Sendai virus-induced expression of IFN-β, ISG52, and ISG56 [7,26,27,28] (Figure 1). Complementing these immune evasion strategies, M protein forms complexes with heat shock protein 70 (HSP70) that modulate both host innate immunity and viral replication efficiency [29].

#### 2.1.3. E Protein

The E protein, PEDV’s smallest structural component (76 amino acids), facilitates virion assembly and budding while exhibiting strategic immune subversion [30]. Localized predominantly in the endoplasmic reticulum (ER), E protein induces ER stress (ERS) by upregulating glucose-regulated protein 78 (GRP78). Concurrently, it activates NF-κB signaling and enhances expression of interleukin-8 (IL-8) and anti-apoptotic Bcl-2—though these events likely manifest during late infection. Contrastingly, early-stage infection features E-mediated suppression of NF-κB activation, potentially sustaining viral replication [31]. As a significant IFN-I antagonist, the PEDV E protein suppresses IFN-I production. Existing evidence indicates that the E protein of PEDV can inhibit the production of IFN-β and ISGs induced by poly(I:C) through blocking the RIG-I-mediated signaling pathway and interfering with the activation of IRF3 [32] (Figure 1). The E protein can also directly bind to IRF3, keeping it in the cytoplasm and blocking its nuclear entry, thereby inhibiting the transcription of IFN-β [33].

Eukaryotic translation initiation is critically regulated at its rate-limiting step by four eIF2α kinases—PKR, PERK, HRI, and GCN2—that phosphorylate eIF2α to control protein synthesis. Central to this process is the eIF4F initiation complex, comprising eIF4E, eIF4G, and eIF4A, with eIF4B and poly(A)-binding protein (PABP) serving as essential cofactors [34]. During PEDV infection, the E protein amplifies expression of GRP78 and calnexin (CANX) while enhancing phosphorylation of PKR and PERK. This dual action activates the PERK/eIF2α arm of the ERS response, consequently suppressing host protein translation [35]. Mechanistically, E-induced eIF2α phosphorylation triggers formation of stress granules (SGs)—translation-silencing ribonucleoprotein aggregates [36]. ER-localized E protein further reorganizes ER architecture into punctate structures and upregulates G3BP1 to promote SG assembly. Crucially, E overexpression causes global translational arrest and endogenous protein synthesis attenuation without altering mRNA transcript levels, confirming translational (not transcriptional) suppression. Collectively, PERK/eIF2α activation is mandatory for both SG biogenesis and translational stasis [32,37].

#### 2.1.4. N Protein

The PEDV N protein, the most abundantly expressed and highly conserved structural protein encoded by PEDV, localizes predominantly in the host cytoplasm where it orchestrates viral RNA transcription/replication and modulates host cell metabolism [38]. Furthermore, N protein employs multifaceted strategies to subvert cellular physiology during host–pathogen conflict. Critically, it prolongs S-phase progression and suppresses host cell proliferation—thereby creating favorable conditions for robust viral replication.

Concomitantly, the N protein disrupts ER homeostasis, triggering accumulation of misfolded proteins that impair ER function and induce ERS. It additionally upregulates IL-8 expression and hijacks autophagy to facilitate viral replication [39]. Crucially, N protein executes coordinated immune evasion by antagonizing both type I (IFN-I) and III interferons (IFN-λ). Mechanistically, it directly binds TANK-binding kinase 1 (TBK1), inhibiting IRF3 phosphorylation and nuclear translocation to suppress IFN-I production [33]. N protein further blocks IFN-β and ISG expression by counteracting IRF3 and NF-κB activation [40], while simultaneously impeding RIG-I/MDA5/MAVS/TRAF3-mediated IFN-β promoter activity [33] (Figure 1). Paradoxically, although N protein activates NF-κB through porcine intestinal TLR2/3/9 pathways, it antagonizes IFN-λ production by blocking NF-κB nuclear translocation [41]. Epigenetically, N-mediated suppression of histone deacetylase 1 (HDAC1) enhances STAT1 acetylation. This post-translational modification inhibits STAT1 phosphorylation, subsequently impairing its nuclear translocation and antiviral gene expression—ultimately enabling immune escape [42].

The structural proteins of PEDV collectively establish a multilayered defense strategy against host innate immunity through concerted action. For instance, the S protein subverts IFN-I production by exploiting the EGFR-JAK2-STAT3 signaling axis and facilitates viral release by inducing apoptosis. The M protein targets interferon regulatory factor 7 (IRF7) and multiple PRRs, thereby disrupting IFN-mediated signaling cascades [26]. Concurrently, the E protein suppresses RIG-I signaling and host protein translation by activating the PERK/eIF2α pathway [32]. The N protein antagonizes both type I and III interferons (IFN-I/λ) by impeding the TBK1-IRF3 and NF-κB pathways [42]. Collectively, these structural proteins target critical nodes of innate immune signaling while also compromising cellular homeostasis (e.g., ER function, cell cycle progression), thereby creating an optimized environment for viral replication.

### 2.2. Role of NSPs in the Escape of PEDV from Host Innate Immunity

#### 2.2.1. NSP1

The 110-amino-acid NSP1, processed from the N-terminus of pp1a, localizes to host mitochondria, ER, and Golgi to facilitate viral gene expression. Among all 21 PEDV proteins, NSP1 constitutes the most potent IFN antagonist [39,43]. Coronaviral NSP1 proteins—conserved in alpha- and beta-coronaviruses as genus-specific markers [44,45]—function as key IFN suppressors despite low sequence identity. Crucially, they share a conserved core domain conferring parallel biological functions [46,47,48]. Mechanistically, SARS-CoV (beta-CoV) NSP1 binds the 40S ribosomal subunit, inactivating translation and triggering endonucleolytic cleavage of host mRNAs [49,50]. It concurrently suppresses antiviral signaling by inhibiting IFN production and innate immunity [51,52]. MERS-CoV NSP1 exhibits analogous host gene suppression [53]. Similarly, PEDV NSP1 antagonizes innate immunity, with its N93/95A mutation significantly attenuating viral fitness: mutant-infected cells exhibit 6.8-fold elevated IFN-β mRNA, enhanced IFN sensitivity, and impaired replication efficiency—demonstrating NSP1’s indispensable role in immune evasion.

**Figure 2:**
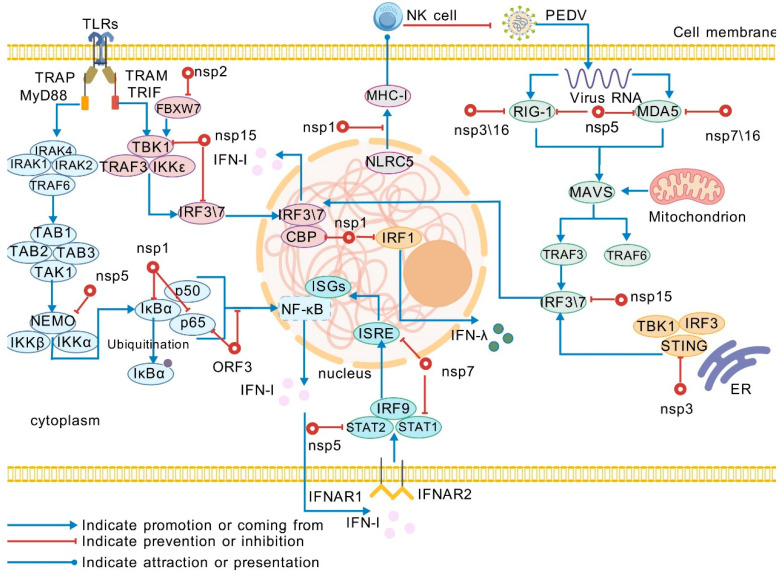
Role of NSPs and the accessory protein in the escape of PEDV from host innate immunity. Upon infecting host cells, PEDV antagonizes the host innate immune response through mechanisms involving its NSPs and accessory protein, thereby facilitating viral evasion of host immune defenses. Following PEDV infection, host cells initiate immune signaling cascades, which are subsequently suppressed primarily by viral NSP family proteins and the ORF3 protein. Viral recognition occurs via TLRs on the cell membrane, activating pathways such as MyD88 and TRIF, while intracellular PRRs including RIG-I and MDA5 detect viral RNA, triggering the MAVS-mediated signaling pathway. The NSPs of PEDV inhibit key signaling molecules like IRFs and NF-κB, disrupting both the production and signaling of IFN-I. Furthermore, these NSPs suppress nuclear antiviral factors, including ISGs. Additionally, PEDV interferes with the MHC-I antigen presentation pathway, further complicating the recognition of infected cells by NK cells. Collectively, these mechanisms strategically enable PEDV to evade the host innate immune systemTLRs: Toll-like receptors; TIRAP: Toll interleukin 1 receptor domain containing adaptor protein; TRAM: Toll-like receptor adaptor molecule; MyD88: myeloid differentiation factor 88; TRIF: TIR-domain-containing adaptor inducing interferon-β; IRAK4: interleukin-1 receptor-associated kinase 4; TAB1: TGF-beta-activated kinase 1 and MAP3K7-binding protein 1; TAK1: transforming growth factor-β-activated; IKKβ: inhibitor of kappa B kinase beta; IKKα: IκB kinase alpha; IκBα: inhibitor of nuclear factor κBα; IKKε: inhibitor of nuclear factor-κb epsilon kinase; ISGs: interferon-stimulated genes; STING: stimulator of interferon genes; ISRE: interferon stimulated response element. This figure was created by BioGDP.com [23].

In vivo neonatal piglet challenge studies demonstrate that the N93/95A mutant confers complete protection against lethal infection, preventing severe watery diarrhea and mortality. These findings establish N93/N95 residues as structural determinants of PEDV virulence and immune evasion, wherein mutations fundamentally alter viral pathogenesis [54]. Previous evidence indicates that the region encompassing residues N93 and N95 in NSP1 may directly participate in binding host-associated proteins [54]. The N93/95A mutation induces structural alterations in this region, impairing its interaction with the CREB-binding protein (CBP). Consequently, this mutation fails to effectively block the formation of the CBP-IRF3 complex, leading to upregulated expression of IFN-I and interferon-stimulated genes (ISGs). These findings demonstrate that residues N93 and N95 play a critical role in NSP1-mediated immune evasion by maintaining proper binding between NSP1 and host proteins, thereby suppressing host immune responses [54,55]. While MAVS primarily drives IFN-I induction and peroxisomes facilitate IFN-λ production via peroxisomal MAVS-dependent signaling—orchestrated by IRF1—NSP1 inhibits IFN-III by blocking IRF1 nuclear translocation and reducing peroxisomal abundance. Beyond IFN antagonism, NSP1 impedes NF-κB nuclear translocation and suppresses expression of IFN-β and proinflammatory cytokines (TNF-α, IL-1β, IL-6, IL-15, IL-17). Early in PEDV-infected LLC-PK1 cells, NSP1 targets IκBα phosphorylation and ubiquitination, preventing p65 nuclear translocation and thereby terminating NF-κB signaling—effectively silencing antiviral cytokine cascades [43] (Figure 2).

Recent evidence demonstrates that PEDV NSP1 selectively suppresses virus-induced MHC-I upregulation by targeting NLRC5—the principal transactivator of MHC-I genes. Notably, NSP1 does not alter basal expression of NLRC5 or MHC-I under physiological conditions, indicating its exclusive inhibition of de novo NLRC5 mRNA translation during viral infection (Figure 2) [56]. This targeted suppression enables infected cells to mimic uninfected phenotypes by reducing MHC-I surface expression, thereby evading surveillance by CD8^+^ T cells and natural killer (NK) cells. Consequently, infected cells escape cytotoxic elimination, facilitating persistent viral infection through impaired adaptive immunity [56].

#### 2.2.2. NSP2

FBXW7 functions as an innate antiviral factor that enhances RIG-I and TBK1 expression while inducing ISGs to elevate host antiviral states. PEDV NSP2 counteracts this defense by interacting with FBXW7 and targeting it for K48-linked ubiquitin-proteasomal degradation, thereby suppressing cellular antiviral immunity [57] (Figure 2). Recent studies further establish NSP2 as a TBK1-targeting virulence determinant. Mechanistically, NSP2 induces macroautophagy and recruits the selective autophagy receptor NBR1 (neighbor of BRCA1 gene 1), which subsequently mediates K48-linked ubiquitination of TBK1 and delivers it to autophagosomes for degradation. This identifies enteric coronavirus NSP2 as a master regulator of innate immunity suppression through NBR1-mediated selective autophagy of TBK1 [58].

#### 2.2.3. NSP3

As the largest transmembrane protein encoded by PEDV genome, NSP3 possesses intricate structural complexity featuring two papain-like protease (PLpro) domains designated PLP1 and PLP2. Previous evidence indicates that PEDV subverts IRF3 activation during infection, thereby blocking double-stranded RNA (dsRNA)-induced IFN-β production. Within this immune evasion paradigm, PLP2 functions as a pivotal IFN antagonist through its deubiquitinase (DUB) activity, which reduces global ubiquitinated protein levels in infected cells. Furthermore, PEDV PLP2 demonstrates stringent substrate specificity—processing both K48- and K63-linked polyubiquitin chains with potent DUB functionality [59].

Beyond these functions, PEDV PLP2 exhibits potent suppressive activity in innate immunity regulation. It physically interacts with RIG-I and STING (as demonstrated by co-immunoprecipitation) and deubiquitinates both molecules. This modification robustly inhibits activation of the RIG-I and STING signaling pathways, consequently curtailing IFN production. Additionally, PEDV-encoded PLPro operates as an immune evasion effector that counteracts ISGs, thereby crippling host antiviral defenses to facilitate persistent viral replication [59,60] (Figure 2). Complementary studies of SARS-CoV and TGEV NSP3 reveal conserved mechanisms whereby NSP3 blocks NF-κB-mediated cytokine responses through inhibition of IκBα ubiquitination plus suppression of p56 phosphorylation and nuclear translocation. Whether PEDV exploits identical pathways for immune evasion remains an open question warranting further investigation [61].

#### 2.2.4. NSP5

NSP5 functions as an essential 3C-like protease (3CLpro) in coronavirus replication, executing indispensable proteolytic processing critical to the viral lifecycle. Given its irreplaceable role in polyprotein maturation, this enzyme is alternatively designated the main protease, and its catalytic activity depends on the catalytic dimer composed of Cys145 and His41 [62,63]. Notably, NSP5 displays exceptional evolutionary conservation across all known coronaviruses, maintaining highly conserved amino acid sequences and tertiary structures [64,65]. Under physiological conditions, the protease operates predominantly as a homodimer that precisely cleaves viral precursor polyproteins pp1a/pp1ab. This processing generates non-structural proteins NSP4-NSP16, which collectively underpin viral structural integrity and replicative functions [63].

The NF-κB essential modulator (NEMO) serves as a pivotal molecule in host innate immunity, orchestrating the activation of NF-κB, IRF3, and IRF7 in response to RNA viruses, thereby governing IFN production. A previous study demonstrates that NEMO not only facilitates MAVS-mediated IKKα/β activation but is also indispensable for optimal TBK1/IKKε phosphorylation—a critical event in innate immune signaling cascades [66].

Notably, PEDV subverts host immune defenses by targeting NEMO through its NSP5. Beyond its well-established role as a cysteine proteinase responsible for most cleavages within the viral polyprotein, NSP5 functions as a potent IFN antagonist that exploits its proteolytic activity to cleave NEMO at glutamine 231 (Q231) during PEDV infection [67,68]. This cleavage site is evolutionarily conserved and critically disrupts NEMO-dependent NF-κB activation required for IFN induction [67]. Concurrently, NSP5 targets transcription factor STAT2 for proteolytic cleavage. These dual cleavage events dismantle NEMO-mediated IFN induction capacity and disrupt IFN-I signaling cascades, culminating in substantially attenuated IFN-I production. This multipronged evasion strategy facilitates PEDV escape from host immune surveillance (Figure 2) [67].

The above-mentioned proteolytic activity represents a key immune evasion strategy employed by PEDV. Concurrently, PEDV NSP5 subverts the RIG-I/MDA5 signaling axis by inhibiting its activation cascade upstream of TBK1 (Figure 2). Critically, as TBK1 serves as the central signaling nexus bridging pathogen recognition and IFN induction, this upstream blockade enables NSP5 to disrupt innate immune responses at their inception phase. By aborting antiviral signaling initiation at the earliest checkpoint, NSP5 creates temporal and spatial advantages for viral replication, assembly, and dissemination within host cells [67,69].

#### 2.2.5. NSP6

As a key NSP orchestrating host autophagy during PEDV infection, NSP6 plays a central role in hijacking host cellular physiology to facilitate viral replication. In porcine intestinal epithelial cells (IPEC-J2), NSP6 suppresses the PI3K/Akt/mTOR signaling axis by downregulating phosphorylation of mTOR and its downstream effector p70S6K, thereby inducing autophagosome formation. Critically, interference with endogenous p53 expression significantly attenuates NSP6-induced autophagy and reduces viral titers, confirming that NSP6-mediated autophagy depends on the PI3K/Akt/mTOR-p53 axis and is essential for efficient PEDV replication in intestinal cells [69,70].

Notably, NSP6’s autophagy induction mechanism exhibits cell type-specificity. In Vero cells, PEDV infection elicits no significant change in mTOR phosphorylation; instead, NSP6 triggers autophagy via activation of the AMPK-ULK1 and JNK pathways, independent of the PI3K/Akt cascade [71]. This divergence may be attributed to baseline activity differences in signaling pathways and distinct expression profiles of NSP6-interacting proteins across cell types, suggesting PEDV flexibly adapts its autophagy regulation strategies to optimize infection in different host microenvironments.

Furthermore, as a component of the coronavirus replication-transcription complex (RTC), NSP6-induced autophagy not only provides energy and substrates for viral replication but also degrades host antiviral proteins (e.g., IRF3, TBK1) to suppress innate immune responses. This establishes a synergistic “autophagy induction-immune suppression-viral replication” loop that enhances PEDV evasion of host defenses [69,72].

#### 2.2.6. NSP7

Comprising 83 amino acids, NSP7 exhibits high sequence conservation across diverse PEDV strains. Subcellular localization studies confirm its predominant cytoplasmic functionality in host cells [73]. As a conserved viral protein, NSP7 plays a critical role in PEDV immune evasion, particularly in subverting host IFN-I responses. Through dual-luciferase reporter assays quantifying promoter activity and qPCR-based gene expression analysis, we demonstrate that NSP7 dose-dependently suppresses IFN-α-induced activation of the IFN-stimulated response element (ISRE) promoter, consequently inhibiting downstream ISG expression [74] (Figure 2).

NSP7 antagonizes IFN-I signaling through a multi-tiered mechanism. It disrupts IFN-α-triggered JAK-STAT signaling, attenuating ISG expression. Paradoxically, immunoblot analyses demonstrate that NSP7 neither alters protein abundance nor phosphorylation kinetics of JAK1, Tyk2, STAT1, or STAT2, nor impedes assembly of the interferon-stimulated gene factor 3 (ISGF3) complex [75]. Crucially, NSP7 blocks nuclear translocation of STAT1/STAT2 by competitively binding STAT1 and disrupting its recognition by karyopherin α1 (KPNA1)—the principal nuclear import adaptor for STAT proteins (Figure 2). This KPNA1 sequestration abrogates ISGF3 nuclear trafficking, ultimately suppressing IFN-I signaling and downstream antiviral gene expression to facilitate viral immune evasion [66,75].

Furthermore, PEDV NSP7 targets MDA5 and interacts with its caspase activation and recruitment domain (CARD), thereby sequestering MDA5 from protein phosphatase 1 (PP1) catalytic subunits PP1α and PP1γ. This interaction blockade suppresses dephosphorylation at MDA5 Ser828, consequently sustaining the inactive state of MDA5 and impairing MDA5-mediated IFN production [76].

#### 2.2.7. NSP10 and NSP14

NSP14 exhibits high conservation across the coronavirus family. All orthologs harbor dual catalytic activities: 3′–5′ exoribonuclease (ExoN) proofreading and N7-methyltransferase (N7-MTase) cap modification. Through concerted ExoN-mediated RNA error correction and N7-MTase-driven RNA cap methylation, this bifunctional enzyme ensures genomic replication fidelity—providing the mechanistic foundation for stable viral propagation and accurate genetic information transmission [77].

Systematic evaluation of NSP14 function via combinatorial overexpression and RNAi strategies in human and porcine cells consistently revealed its dose-dependent reduction of RIG-I protein levels without altering mRNA abundance, indicating post-translational regulation. Cycloheximide-chase assays demonstrated that NSP14 shortened RIG-I half-life from approximately 6 h to 2 h. This degradation was fully blocked by proteasome inhibitor MG132 but unaffected by lysosomal inhibitors, explicitly implicating the ubiquitin-proteasome pathway [78]. Co-immunoprecipitation, co-localization, and truncation mutagenesis established direct binding between the N-terminal 1–180 aa domain of NSP14 and the CARD of RIG-I, providing the structural basis for degradation. Functionally, dual-luciferase reporter assays showed that NSP14 abrogated RIG-I-mediated IFN-β promoter activity by ~70% [78]. As a crucial PRR, RIG-I normally detects PEDV RNA to activate the MAVS pathway, thereby promoting IRF3 phosphorylation and nuclear translocation to induce IFN-I production and establish an antiviral state [79,80]. However, NSP14-mediated degradation of RIG-I markedly diminishes the host cell’s capacity to recognize PEDV RNA, impairing MAVS signaling activation and disrupting IRF3-dependent transcriptional initiation of IFN-I. Consequently, the host fails to mount timely early antiviral responses, which operates through two distinct mechanisms: (i) it reduces direct suppression of viral replication by IFN-I-induced ISGs, which inhibit viral RNA synthesis and disrupt viral protein translation; and (ii) it attenuates IFN-I-mediated bystander cell alerting, preventing neighboring cells from entering a preemptive antiviral state. Collectively, these effects create a critical temporal window and permissive environment that facilitates extensive PEDV replication, assembly, and cell-to-cell dissemination within infected tissues. This cascade ultimately disrupts the host–virus equilibrium, favoring viral persistence [78]. And, compared to wild-type virus, an NSP14-deficient PEDV mutant elicited 2.3-fold higher IFN-β induction and exhibited a 1-log reduction in viral titer. Collectively, NSP14 facilitates viral immune evasion by targeting RIG-I for proteasomal degradation to suppress the IFN-I pathway [78].

The 133-amino-acid NSP10 protein (≈14.6 kDa) adopts a finely resolved tertiary structure characterized by an N-terminal antiparallel α-helical pair forming a compact core, a central irregular β-sheet imparting structural plasticity, and a C-terminal domain harboring two zinc-finger motifs with extensive coil regions that collectively underpin its functionality [80]. Within the coronavirus RTC, NSP10 cooperates with NSP14, NSP16, and other replicase subunits to maintain genomic fidelity. Specifically, NSP10 allosterically stimulates NSP14’s 3′–5′ exoribonuclease (ExoN) activity and NSP16’s 2′-O-methyltransferase function during viral RNA synthesis—enhancing replication accuracy to ensure precise genetic information transmission [81]. Structural and biochemical analyses confirm that NSP10 and NSP14 persistently interact as a heterodimeric complex, with domain-specific binding stabilizing and potentiating NSP14’s catalytic efficiency [82]. Functional studies establish NSP10 as an essential cofactor for viral RNA synthesis, wherein its binding to NSP14 elevates ExoN proofreading activity by >35-fold and accelerates misincorporated nucleotide excision [83,84].

#### 2.2.8. NSP15

As a coronavirus endoribonuclease, NSP15 targets TBK1 and IRF3 RNAs for degradation via its endonucleolytic activity during viral RNA synthesis. Given the central roles of TBK1 and IRF3 in host IFN regulation, their diminished RNA levels directly suppress IFN production and substantially reduce ISG induction, thereby enabling PEDV to subvert the host innate immune response [85] (Figure 2). A study has shown that the residues H226A, H241A and K282A are crucial for the ribonuclease activity of PEDV NSP15 [85]. Through large-scale screening using dual-luciferase reporter systems, researchers identified NSP15 as a potent suppressor of both poly(I:C)-induced IFN-β and IFN-λ activation—a discovery that pinpointed key mechanisms underpinning PEDV immune evasion [43,55]. Subsequent reverse genetics studies employing infectious clones with rigorous controls demonstrated that NSP15’s antagonism of host IFN-I (IFN-β) and IFN-III (IFN-λ) responses strictly requires its endoribonuclease activity, definitively establishing the molecular basis for its function [86].

In 2021, Gao et al. demonstrated that infectious bronchitis virus (IBV) NSP15 evades protein kinase recognition and suppresses SG formation by reducing dsRNA accumulation in host cells [87]. Mechanistically, EndoU ribonuclease activity proved essential for SG suppression. Parallel studies in PEDV revealed that NSP15 overexpression in LLC-PK1 cells significantly inhibited both eIF2α-dependent and -independent SG assembly. Consistently, coronavirus NSP15 expression universally blocked PKR-eIF2α-SG signaling across multiple cell models. Given the catalytic core domain’s high conservation among coronaviruses, these findings indicate NSP15 mediates broadly conserved suppression of host antiviral responses [87].

#### 2.2.9. NSP16

Within the intricate gene expression and regulation network of coronaviruses, NSP16 emerges as an essential component of the viral life cycle due to its distinctive functionality and exceptional conservation. This 2′-O-methyltransferase (MTase) plays a critical role in viral RNA synthesis. Sequence analyses across multiple coronaviruses reveal exceptionally high amino acid conservation in NSP16 among divergent strains—indicating its indispensable and evolutionarily conserved function in viral survival and transmission [88].

Studies on PEDV-mediated IFN antagonism reveal that NSP16 acts as a potent immune evasion factor during virus–host interactions. Mechanistically, NSP16 exploits its methyltransferase activity to suppress IRF3 phosphorylation—a pivotal node in innate immune signaling. Impaired IRF3 phosphorylation directly blocks RIG-I/MDA5-triggered signaling cascades (Figure 2), enabling PEDV to evade host immune surveillance and counteract antiviral responses. Structural and functional analyses further demonstrate that NSP16’s conserved KDKE motif serves as the critical domain suppressing IFN-β and ISRE promoter activation, providing mechanistic insights into its immune subversion function. Mutagenesis replacing catalytic aspartate residues with alanine ablates methyltransferase activity, substantially diminishing these immune-subversive functions [89,90].

Studies demonstrate that NSP16 significantly downregulates RIG-I/MDA5-mediated IFN-β and ISRE activity, thereby attenuating host antiviral immunity [90]. Correspondingly, NSP16 overexpression markedly suppresses mRNA levels of IFIT family members (IFIT1, IFIT2, IFIT3)—key antiviral effectors whose inhibited expression confirms NSP16’s immunosuppressive function [90]. Notably, within PEDV’s regulatory network, NSP10 enhances NSP16-mediated IFN-β suppression, revealing coordinated immune evasion. As a central transcription factor in the IFN-I production pathway, IRF3 requires phosphorylation for nuclear translocation and binding to the IFN-β promoter. NSP16 directly inhibits IRF3 phosphorylation, thereby preventing its nuclear entry and subsequent IFN-β gene transcription, which significantly reduces IFN-β secretion by host cells [90]. From the viral perspective, diminished IFN-β levels alleviate immune pressure during replication: the viral RNA polymerase escapes suppression by IFN-β-induced ISGs, enabling efficient viral genome replication, while viral protein translation proceeds unhindered by ISG interference, enhancing virion assembly efficiency. Conversely, from the host standpoint, insufficient IFN-β fails to activate antiviral gene expression in neighboring cells, rendering them susceptible to PEDV infection and expanding viral dissemination within the host. Furthermore, NSP16-mediated downregulation of RIG-I/MDA5-driven IFN-β activation disrupts the host’s dual defense mechanism involving multiple PRRs, promoting stable viral colonization in host cells and facilitating subsequent virion release and secondary infection [69,90]. This coordinated suppression exemplifies an evolved viral strategy to subvert innate immunity, with NSP16 playing a pivotal role in establishing a permissive intracellular environment for PEDV persistence. Both methyltransferases NSP14 and NSP16 antagonize innate immunity; however, comparative analyses indicate NSP16 more effectively modulates immune-related gene expression and precisely regulates host responses [90,91].

#### 2.2.10. Other NSPs

PEDV NSP6 induces autophagy and promotes viral replication in porcine IECs via the PI3K/Akt/mTOR signaling pathway [69]. As a key component of coronavirus RNA replication machinery, NSP8 forms a hexadecameric complex with NSP7 that mediates nucleic acid binding to NSP12, constituting the minimal RNA polymerase complex [92,93]. NSP8 exhibits high RNA-dependent polymerase activity exclusively when assembled with both NSP7 and NSP12 at identical temperatures [84]. Additionally, PEDV NSP8 suppresses IFN-III activity by reducing IRF1 promoter activity in vitro. Reconstitution studies using infectious clones attempted—yet failed—to rescue viable virus upon rearranging NSP7 and NSP8 genes. This work and accumulating evidence establish NSP7 and NSP8 as essential cofactors for viral replication, suggesting their potential role in PEDV-mediated innate immune suppression despite unresolved mechanistic details [94,95].

The nonstructural proteins of PEDV function as core effector molecules for innate immune evasion, with each protein targeting distinct nodes of the host antiviral signaling cascade. Collectively, they constitute a coordinated network that not only suppresses IFN production and blocks IFN signaling but also evades detection by PRRs. This multifaceted strategy is pivotal for PEDV to establish persistent infection in host cells.

### 2.3. Role of the Accessory Protein ORF3 in the Escape of PEDV from Host Innate Immunity

As the sole identified accessory protein in PEDV, the ∼25-kDa ORF3 critically modulates viral virulence and replication. ORF3 localizes predominantly in the cytoplasm, with partial distribution to the ER and Golgi apparatus. It colocalizes and interacts with the S protein in perinuclear regions and vesicular structures of infected cells, collectively regulating viral replication [96]. ORF3 suppresses both IFN-I (IFN-β) and IFN-III (IFN-λ1) production, though the precise inhibitory mechanisms await further validation [43,55]. Ye et al. established ORF3-stably expressing Vero cells to investigate its subcellular localization and host interactions, concurrently assessing its impact on virion production. Results demonstrate cytoplasmic ORF3 localization, S-phase prolongation that disrupts cell cycle progression, and increased vesicle formation compared to native Vero cells. Notably, attenuated PEDV replicates more efficiently than virulent strains in ORF3-expressing cells [97].

Furthermore, ORF3 exerts dual regulation on viral replication through apoptosis and autophagy pathways: it directly suppresses infected-cell apoptosis to enhance viral proliferation, while concurrently promoting LC3-I-to-LC3-II conversion to induce autophagic flux. Accumulating in the ER, ORF3 upregulates GRP78 expression and activates the PERK–eIF2α pathway, thereby triggering ERS. This initiates the unfolded protein response (UPR)—a conserved mechanism restoring proteostasis—ultimately inducing cell death and autophagy [98]. ORF3 also antagonizes host innate immunity by modulating NF-κB signaling, specifically through: (i) inhibiting phosphorylation of IκBα and nuclear factor p65, and (ii) impairing p65 nuclear translocation (Figure 2), which collectively reduce proinflammatory cytokine production (e.g., IL-6, IL-8) [99]. Paradoxically, while ORF3 enhances IκBβ-mediated NF-κB promoter activity, it counterintuitively suppresses IκBβ-driven IFN-β promoter activation and mRNA expression. Overexpression experiments confirm ORF3’s capacity to inhibit poly(I:C)-induced IFN-I production [100].

## 3. Effect of Autophagy on PEDV Escape from Host Innate Immunity

Viral infection of target cells typically induces cellular autophagy and programmed cell death. While viruses exploit the host ER for folding nascent viral proteins, excessive accumulation of unfolded proteins triggers ERS, consequently inducing autophagy. Lin et al. demonstrated that treatment of PEDV-infected IPEC-J2 cells with 100 nM rapamycin—an mTOR pathway inhibitor that induces autophagy—enhances PEDV replication, confirming autophagy’s proviral role [69].

### 3.1. PEDV Induces Autophagy Through ERS

ERS represents a critical cellular stress response mechanism wherein the UPR processes misfolded proteins within the ER lumen. This quality control system initiates multifaceted regulatory measures upon detecting protein misfolding, including attenuation of further protein translocation into the ER to prevent luminal overcrowding—thereby maintaining proteostasis. From a virological perspective, viruses typically exploit the host ER for folding nascent viral proteins post-synthesis. However, excessive accumulation of unfolded proteins in the ER lumen triggers ERS through overload-induced signaling [101]. Cellular autophagy—a lysosome-dependent degradation pathway—constitutes another essential biological process inducible by diverse stimuli, including ERS. During ERS activation, autophagy is upregulated to eliminate aberrant proteins and damaged organelles, thus preserving cellular homeostasis.

In a series of investigations into PEDV infection mechanisms, Xu et al. first infected host cells with PEDV and subsequently detected substantial accumulation of viral structural proteins E and N within the ER [31]. This observation prompted investigations into whether the subcellular localization of these essential structural components correlates with host physiological alterations. To address this, the team designed experiments examining the relationship between E/N proteins and ERS. Results demonstrated that both proteins significantly induce ERS in host cells—a conclusion robustly supported by multiple experimental datasets. Given the ER’s critical role in protein synthesis, processing, and functional homeostasis, these findings indicate that during PEDV infection, E and N proteins likely disrupt ER functionality by triggering ERS, thereby impairing cellular physiology. This mechanism provides key insights into how PEDV manipulates host cell processes to facilitate infection [31].

Zou et al. uncovered a notable mechanism wherein ORF3 protein—localized predominantly in the ER during PEDV infection—elevates GRP78 expression to activate the PERK-eIF2α signaling axis, thereby inducing ERS [98]. Furthermore, this viral protein triggers autophagy by promoting LC3-I to LC3-II conversion, a critical autophagosome formation step. Crucially, pharmacological inhibition of ERS using 4-PBA significantly suppressed LC3 lipidation, demonstrating that ORF3-induced autophagy is mechanistically dependent on ERS activation [98].

### 3.2. The Regulation of Autophagy by PEDV-Related Proteins

Beyond NSP6-mediated modulation of host immune pathways for autophagy-dependent evasion of innate immunity, PEDV deploys multiple viral proteins—including the N protein, NSP2, and ORF3—to co-opt autophagy through distinct mechanisms [69]. The N protein induces ERS, indirectly triggering autophagy while exploiting this process to degrade antiviral host proteins (e.g., IRF3, TBK1) that suppress IFN-β production [33,39]. NSP2 recruits the selective autophagy receptor NBR1 to mediate autophagic degradation of TBK1, thereby dismantling key innate immune signaling hubs [58]. ORF3 activates the PERK-eIF2α axis to drive ERS-associated autophagy, a process strictly dependent on GRP78 upregulation [98]. Collectively, these proteins orchestrate a synergistic autophagy-dependent immune evasion network by targeting discrete nodes of the autophagic pathway. However, their precise interplay and signaling cross-talk warrant further investigation to elucidate PEDV’s systemic subversion of host defenses.

## 4. PEDV Evades the Host Innate Immunity by Hiding PAMPs

During virus–host interplay, viruses evolve sophisticated evasion tactics to preserve genomic integrity and ensure productive infection. Key strategies include viral endoribonuclease activity and 5′ cap structures that shield viral RNA from host recognition and degradation [102,103]. Furthermore, the N7-MTase activity of non-structural protein NSP14 is indispensable for viral transcription/translation while simultaneously subverting host defenses—specifically preventing PRRs from initiating innate immune responses against viral mRNA by masking it as “self” [104].

PEDV NSP15 possesses endoribonuclease activity that enables immune evasion by cleaving viral dsRNA, thereby reducing accumulation and preventing recognition by host sensors MDA5 and PKR. Crucially, NSP15’s EndoU catalytic function exhibits context-dependent plasticity—modulated by dsRNA secondary structures and post-transcriptional modifications—that dynamically adapts to intracellular conditions. This structural flexibility allows NSP15 to suppress dsRNA sensor activation, ultimately facilitating persistent viral replication [86,105,106,107,108].

PEDV NSP16, a methyltransferase-family enzyme, catalyzes viral RNA cap modification. This biochemical mimicry renders viral RNA structurally indistinguishable from host mRNA, effectively evading MDA5 surveillance and reducing antiviral immune activation. During RNA virus replication, inevitable generation of dsRNA and 5′-triphosphate RNA typically triggers PRR-mediated immune responses. However, through coordinated activity of NSPs including NSP15 and NSP16, PEDV subverts host immunosurveillance to ensure sustained viral replication and dissemination [89,90,109,110].

## 5. Other Pathways Through Which PEDV Evades the Host Innate Immunity

The MAPK signaling pathway orchestrates cellular responses to intra- and extracellular stimuli through key mediators ERK, JNK, and p38. During PEDV infection, ERK and p38 activation paradoxically enhances viral replication without inducing host cell cycle arrest or apoptosis [111]. Guo et al. demonstrated that PEDV exploits the ubiquitin-proteasome pathway to degrade phosphorylated STAT1 (p-STAT1), thereby suppressing IFN signaling [112]. Furthermore, infection triggers caspase-8-mediated cleavage of Ras-GTPase-activating protein-binding protein 1 and disrupts SG assembly to facilitate viral replication [113]. PEDV also degrades partitioning defective 3 (PARD3)—a scaffold protein maintaining epithelial tight junctions—via proteasome-dependent mechanisms to promote viral proliferation [114]. Crucially, HSP27 normally potentiates antiviral responses by activating NF-κB to induce IFN-β and downstream ISGs. However, PEDV suppresses HSP27 function in Marc-145 cells, attenuating IFN-β and ISG expression to evade host immunity [115].

## 6. Closing Remarks

Significant progress has been made in recent years regarding PEDV evasion of the host innate immune system. Research has revealed that PEDV employs multiple sophisticated mechanisms to subvert host immune surveillance or antagonize innate immune responses. These include directly or indirectly inhibiting host IFN production, concealing its PAMPs to avoid recognition by host PRRs, and attenuating inflammatory responses through pathways such as NF-κB signaling. Furthermore, PEDV exploits host cellular physiological processes, including autophagy, ERS, apoptosis, and various host cell signaling pathways, to facilitate viral replication and evade innate immunity.

While current research has unveiled partial mechanisms of PEDV immune regulation, significant knowledge gaps remain in the field, which simultaneously delineate promising directions for future investigations. On the one hand, the hierarchical contribution of distinct immune evasion mechanisms in vivo remains undetermined. For instance, whether E protein-mediated ERS, IL-8 expression regulation, NF-κB signaling modulation, or other potential unknown strategies play dominant roles in viral infection and dissemination during natural infection requires validation through dynamic tracking experiments in animal models. On the other hand, whether synergistic or antagonistic effects exist among viral proteins (e.g., NSP10 enhancing NSP14 activity to cooperatively inhibit IFN responses), thereby forming more efficient immune evasion networks, constitutes an unresolved puzzle. Furthermore, the impact of host heterogeneity on immune evasion remains unclear—would the evasion efficiency of PEDV be altered by differential expression of immune molecules (such as the expression levels of RIG-I and IRF3) depending on the host’s age, breed, or the different infection sites? The answers to these questions require the establishment of more realistic infection models in future research.

From a technical perspective, multi-omics integration analysis may serve as a pivotal solution. By combining transcriptomic, proteomic, and metabolomic data, this approach could systematically identify core regulatory nodes in host immune pathways post-PEDV infection, while uncovering novel virus–host interaction targets. Super-resolution imaging techniques (e.g., STED, SIM) would enable direct visualization of in situ binding dynamics between viral proteins and host molecules, elucidating the mechanistic impact of subcellular localization on immune regulation. Additionally, targeted intervention experiments based on known interaction sites could functionally validate critical mechanisms, providing direct evidence for developing novel prevention and control strategies.

In conclusion, future research should adopt a “mechanistic elucidation—technical validation—translational application” framework. Through continuous discovery and resolution of unanswered questions, not only can our understanding of how PEDV breaks through the host’s innate immune barrier be deepened, but it can also lay the foundation for designing more targeted and adaptable countermeasures (such as multi-epitope vaccines and immunomodulators). Ultimately, these efforts will contribute to safeguarding animal health and promoting sustainable development in the livestock industry.

## Data Availability

Not applicable.

## References

[1] Koonpaew S., Teeravechyan S., Frantz P.N., Chailangkarn T., Jongkaewwattana A. (2019). PEDV and PDCoV Pathogenesis: The Interplay Between Host Innate Immune Responses and Porcine Enteric Coronaviruses. Front. Vet. Sci..

[2] Cavanagh D.J.V. (1996). Nidovirales: A new order comprising Coronaviridae and Arteriviridae. Arch. Virol..

[3] Kocherhans R., Bridgen A., Ackermann M., Tobler K. (2001). Completion of the porcine epidemic diarrhoea coronavirus (PEDV) genome sequence. Virus Genes..

[4] Turvey S.E., Broide D.H. (2010). Innate immunity. J. Allergy Clin. Immunol..

[5] Marshall J.S., Warrington R., Watson W., Kim H.L. (2018). An introduction to immunology and immunopathology. Allergy Asthma Clin. Immunol..

[6] Wang R., Lan C., Benlagha K., Camara N.O.S., Miller H., Kubo M., Heegaard S., Lee P., Yang L., Forsman H. (2024). The interaction of innate immune and adaptive immune system. MedComm.

[7] Yang J., Zhu Z., Zheng H. (2020). Porcine Epidemic Diarrhea Virus and the Host Innate Immune Response. Pathogens.

[8] Sun D.B., Feng L., Shi H.Y., Chen J.F., Liu S.W., Chen H.Y., Wang Y.F. (2007). Spike protein region (aa 636789) of porcine epidemic diarrhea virus is essential for induction of neutralizing antibodies. Acta Virol..

[9] Reguera J., Santiago C., Mudgal G., Ordoño D., Enjuanes L., Casasnovas J.M. (2012). Structural bases of coronavirus attachment to host aminopeptidase N and its inhibition by neutralizing antibodies. PLoS Pathog..

[10] Eckert D.M., Kim P.S. (2001). Mechanisms of viral membrane fusion and its inhibition. Annu. Rev. Biochem..

[11] Li F. (2016). Structure, Function, and Evolution of Coronavirus Spike Proteins. Annu. Rev. Virol..

[12] Li C., Li W., Lucio De Esesarte E., Guo H., Van Den Elzen P., Aarts E., Van Den Born E., Rottier P.J.M., Bosch B.J. (2017). Cell Attachment Domains of the Porcine Epidemic Diarrhea Virus Spike Protein Are Key Targets of Neutralizing Antibodies. J. Virol..

[13] Chang C.Y., Cheng I.C., Chang Y.C., Tsai P.S., Lai S.Y., Huang Y.L., Jeng C.R., Pang V.F., Chang H.W. (2019). Identification of Neutralizing Monoclonal Antibodies Targeting Novel Conformational Epitopes of the Porcine Epidemic Diarrhoea Virus Spike Protein. Sci. Rep..

[14] Wang X., Wang L., Huang X., Ma S., Yu M., Shi W., Qiao X., Tang L., Xu Y., Li Y. (2017). Oral Delivery of Probiotics Expressing Dendritic Cell-Targeting Peptide Fused with Porcine Epidemic Diarrhea Virus COE Antigen: A Promising Vaccine Strategy against PEDV. Viruses.

[15] Sun D., Feng L., Shi H., Chen J., Cui X., Chen H., Liu S., Tong Y., Wang Y., Tong G. (2008). Identification of two novel B cell epitopes on porcine epidemic diarrhea virus spike protein. Vet. Microbiol..

[16] Okda F.A., Lawson S., Singrey A., Nelson J., Hain K.S., Joshi L.R., Christopher-Hennings J., Nelson E.A., Diel D.G. (2017). The S2 glycoprotein subunit of porcine epidemic diarrhea virus contains immunodominant neutralizing epitopes. Virology.

[17] Cruz D.J., Kim C.J., Shin H.J. (2008). The GPRLQPY motif located at the carboxy-terminal of the spike protein induces antibodies that neutralize Porcine epidemic diarrhea virus. Virus Res..

[18] Xu M., Yang Z., Yang N., Li H., Ma H., Yi J., Hou H., Han F., Ma Z., Chen C. (2025). Development and Immunogenicity Study of Subunit Vaccines Based on Spike Proteins of Porcine Epidemic Diarrhea Virus and Porcine Transmissible Gastroenteritis Virus. Vet. Sci..

[19] Li M., Sun X., Chen Y., Wang S., Li Q., Wang Y., Wang Y., Li R., Ding P., Zhang G. (2024). Enhancing humoral and mucosal immune response of PED vaccine candidate by fusing S1 protein to nanoparticle multimerization. Vet. Microbiol..

[20] Yan Q., Liu X., Sun Y., Zeng W., Li Y., Zhao F., Wu K., Fan S., Zhao M., Chen J. (2022). Swine Enteric Coronavirus: Diverse Pathogen-Host Interactions. Int. J. Mol. Sci..

[21] Yang L., Xu J., Guo L., Guo T., Zhang L., Feng L., Chen H., Wang Y., Pfeiffer J.K. (2018). Porcine epidemic diarrhea virus-induced epidermal growth factor receptor activation impairs the antiviral activity of type I interferon. J. Virol..

[22] Chen Y., Zhang Z., Li J., Gao Y., Zhou L., Ge X., Han J., Guo X., Yang H. (2018). Porcine epidemic diarrhea virus S1 protein is the critical inducer of apoptosis. Virol. J..

[23] Jiang S., Li H., Zhang L., Mu W., Zhang Y., Chen T., Wu J., Tang H., Zheng S., Liu Y. (2025). Generic Diagramming Platform (GDP): A comprehensive database of high-quality biomedical graphics. Nucleic Acids Res..

[24] Xu X.G., Zhang H.L., Zhang Q., Dong J., Huang Y., Tong D.W. (2015). Porcine epidemic diarrhea virus M protein blocks cell cycle progression at S-phase and its subcellular localization in the porcine intestinal epithelial cells. Acta Virol..

[25] Wang R., Yu R., Chen B., Si F., Wang J., Xie C., Men C., Dong S., Li Z. (2020). Identification of host cell proteins that interact with the M protein of porcine epidemic diarrhea virus. Veter. Microbiol..

[26] Li S., Zhu Z., Yang F., Cao W., Yang J., Ma C., Zhao Z., Tian H., Liu X., Ma J. (2021). Porcine Epidemic Diarrhea Virus Membrane Protein Interacted with IRF7 to Inhibit Type I IFN Production during Viral Infection. J. Immunol..

[27] Lui P.Y., Wong L.Y., Fung C.L., Siu K.L., Yeung M.L., Yuen K.S., Chan C.P., Woo P.C., Yuen K.Y., Jin D.Y. (2016). Middle East respiratory syndrome coronavirus M protein suppresses type I interferon expression through the inhibition of TBK1-dependent phosphorylation of IRF3. Emerg. Microbes Infect..

[28] Chen P., Li S., Zhu Z., Zheng H., Zheng H., Xu Z. (2019). Inhibitory effect of PEDV M protein on type Ⅰ interferon signaling pathway. J. Northwest A F Univ..

[29] Park J.Y., Ryu J., Park J.E., Hong E.J., Shin H.J. (2021). Heat shock protein 70 could enhance porcine epidemic diarrhoea virus replication by interacting with membrane proteins. Vet. Res..

[30] Sun M., Ma J., Yu Z., Pan Z., Lu C., Yao H. (2017). Identification of two mutation sites in spike and envelope proteins mediating optimal cellular infection of porcine epidemic diarrhea virus from different pathways. Vet. Res..

[31] Xu X., Zhang H., Zhang Q., Dong J., Tong D. (2013). Porcine epidemic diarrhea virus E protein causes endoplasmic reticulum stress and up-regulates interleukin-8 expression. Virol. J..

[32] Zheng L., Wang X., Guo D., Cao J., Cheng L., Li X., Zou D., Zhang Y., Xu J., Wu X. (2021). Porcine epidemic diarrhea virus E protein suppresses RIG-I signaling-mediated interferon-β production. Vet. Microbiol..

[33] Ding Z., Fang L., Jing H., Zeng S., Wang D., Liu L., Zhang H., Luo R., Chen H., Xiao S. (2014). Porcine epidemic diarrhea virus nucleocapsid protein antagonizes beta interferon production by sequestering the interaction between IRF3 and TBK1. J. Virol..

[34] Rojas M., Vasconcelos G., Dever T.E. (2015). An eIF2α-binding motif in protein phosphatase 1 subunit GADD34 and its viral orthologs is required to promote dephosphorylation of eIF2α. Proc. Natl. Acad. Sci. USA.

[35] Bartoszewska S., Collawn J.F. (2020). Unfolded protein response (UPR) integrated signaling networks determine cell fate during hypoxia. Cell Mol. Biol. Lett..

[36] Lee J.I., Namkoong S. (2022). Stress granules dynamics: Benefits in cancer. BMB Rep..

[37] Zheng L., Yang Y., Han Y., Yu J., Wu Z., Kay M., Xia W., Chen Z., Ma J., Yang X. (2024). Porcine epidemic diarrhea virus E protein induces formation of stress granules and attenuates protein translation through activation of the PERK/eIF2α signaling pathway. Vet. Microbiol..

[38] Chang C.K., Sue S.C., Yu T.H., Hsieh C.M., Tsai C.K., Chiang Y.C., Lee S.J., Hsiao H.H., Wu W.J., Chang C.F. (2005). The dimer interface of the SARS coronavirus nucleocapsid protein adapts a porcine respiratory and reproductive syndrome virus-like structure. FEBS Lett..

[39] Xu X., Zhang H., Zhang Q., Huang Y., Dong J., Liang Y., Liu H.J., Tong D. (2013). Porcine epidemic diarrhea virus N protein prolongs S-phase cell cycle, induces endoplasmic reticulum stress, and up-regulates interleukin-8 expression. Vet. Microbiol..

[40] Tan Y.W., Fang S., Fan H., Lescar J., Liu D.X. (2006). Amino acid residues critical for RNA-binding in the N-terminal domain of the nucleocapsid protein are essential determinants for the infectivity of coronavirus in cultured cells. Nucleic Acids Res..

[41] Shan Y., Liu Z.Q., Li G.W., Chen C., Luo H., Liu Y.J., Zhuo X.H., Shi X.F., Fang W.H., Li X.L. (2018). Nucleocapsid protein from porcine epidemic diarrhea virus isolates can antagonize interferon-λ production by blocking the nuclear factor-κB nuclear translocation. J. Zhejiang Univ. B.

[42] Xu J., Gao Q., Zhang W., Zheng J., Chen R., Han X., Mao J., Shan Y., Shi F., He F. (2023). Porcine Epidemic Diarrhea Virus Antagonizes Host IFN-λ-Mediated Responses by Tilting Transcription Factor STAT1 toward Acetylation over Phosphorylation To Block Its Activation. mBio.

[43] Zhang Q., Ke H., Blikslager A., Fujita T., Yoo D.J. (2018). Type III Interferon Restriction by Porcine Epidemic Diarrhea Virus and the Role of Viral Protein nsp1 in IRF1 Signaling. J. Virol..

[44] Narayanan K., Ramirez S.I., Lokugamage K.G., Makino S. (2015). Coronavirus nonstructural protein 1: Common and distinct functions in the regulation of host and viral gene expression. Virus Res..

[45] Snijder E.J., Bredenbeek P.J., Dobbe J.C., Thiel V., Ziebuhr J., Poon L.L., Guan Y., Rozanov M., Spaan W.J., Gorbalenya A.E. (2003). Unique and conserved features of genome and proteome of SARS-coronavirus, an early split-off from the coronavirus group 2 lineage. J. Mol. Biol..

[46] Almeida M.S., Johnson M.A., Wüthrich K. (2006). NMR assignment of the SARS-CoV protein nsp1. J. Biomol. NMR.

[47] Jansson A.M. (2013). Structure of alphacoronavirus transmissible gastroenteritis virus nsp1 has implications for coronavirus nsp1 function and evolution. J. Virol..

[48] Shen Z., Ye G., Deng F., Wang G., Cui M., Fang L., Xiao S., Fu Z.F., Peng G. (2018). Structural Basis for the Inhibition of Host Gene Expression by Porcine Epidemic Diarrhea Virus nsp1. J. Virol..

[49] Kamitani W., Huang C., Narayanan K., Lokugamage K.G., Makino S. (2009). A two-pronged strategy to suppress host protein synthesis by SARS coronavirus Nsp1 protein. Nat. Struct. Mol. Biol..

[50] Kamitani W., Narayanan K., Huang C., Lokugamage K., Ikegami T., Ito N., Kubo H., Makino S. (2006). Severe acute respiratory syndrome coronavirus nsp1 protein suppresses host gene expression by promoting host mRNA degradation. Proc. Natl. Acad. Sci. USA.

[51] Narayanan K., Huang C., Lokugamage K., Kamitani W., Ikegami T., Tseng C.T., Makino S. (2008). Severe acute respiratory syndrome coronavirus nsp1 suppresses host gene expression, including that of type I interferon, in infected cells. J. Virol..

[52] Wathelet M.G., Orr M., Frieman M.B., Baric R.S. (2007). Severe acute respiratory syndrome coronavirus evades antiviral signaling: Role of nsp1 and rational design of an attenuated strain. J. Virol..

[53] Nakagawa K., Narayanan K., Wada M., Popov V.L., Cajimat M., Baric R.S., Makino S. (2018). The Endonucleolytic RNA Cleavage Function of nsp1 of Middle East Respiratory Syndrome Coronavirus Promotes the Production of Infectious Virus Particles in Specific Human Cell Lines. J. Virol..

[54] Niu X., Kong F., Xu J., Liu M., Wang Q. (2022). Mutations in Porcine Epidemic Diarrhea Virus nsp1 Cause Increased Viral Sensitivity to Host Interferon Responses and Attenuation In Vivo. J. Virol..

[55] Zhang Q., Shi K., Yoo D. (2016). Suppression of type I interferon production by porcine epidemic diarrhea virus and degradation of CREB-binding protein by nsp1. Virology.

[56] Liu X., Zhang M., Yin L., Kang L., Luo Y., Wang X., Ren L., Zhang G., Yao Y., Liu P. (2024). PEDV evades MHC-I-related immunity through nsp1-mediated NLRC5 translation inhibition. J. Virol..

[57] Li M., Wu Y., Chen J., Shi H., Ji Z., Zhang X., Shi D., Liu J., Tian J., Wang X. (2022). Innate Immune Evasion of Porcine Epidemic Diarrhea Virus through Degradation of the FBXW7 Protein via the Ubiquitin-Proteasome Pathway. J. Virol..

[58] Jiao Y., Zhao P., Xu L.D., Yu J.Q., Cai H.L., Zhang C., Tong C., Yang Y.L., Xu P., Sun Q. (2024). Enteric coronavirus nsp2 is a virulence determinant that recruits NBR1 for autophagic targeting of TBK1 to diminish the innate immune response. Autophagy.

[59] Xing Y., Chen J., Tu J., Zhang B., Chen X., Shi H., Baker S.C., Feng L., Chen Z. (2013). The papain-like protease of porcine epidemic diarrhea virus negatively regulates type I interferon pathway by acting as a viral deubiquitinase. J. Gen. Virol..

[60] Mielech A.M., Chen Y., Mesecar A.D., Baker S.C. (2014). Nidovirus papain-like proteases: Multifunctional enzymes with protease, deubiquitinating and deISGylating activities. Virus Res..

[61] Wang Y., Sun A., Sun Y., Zhang S., Xia T., Guo T., Hao Z., Sun L., Jiang Y., Qiao X. (2019). Porcine transmissible gastroenteritis virus inhibits NF-κB activity via nonstructural protein 3 to evade host immune system. Virol. J..

[62] Chen J., Li Z., Guo J., Xu S., Zhou J., Chen Q., Tong X., Wang D., Peng G., Fang L. (2022). SARS-CoV-2 nsp5 Exhibits Stronger Catalytic Activity and Interferon Antagonism than Its SARS-CoV Ortholog. J. Virol..

[63] Ye G., Deng F., Shen Z., Luo R., Zhao L., Xiao S., Fu Z.F., Peng G. (2016). Structural basis for the dimerization and substrate recognition specificity of porcine epidemic diarrhea virus 3C-like protease. Virology.

[64] Ma M., Yang Y., Wu L., Zhou L., Shi Y., Han J., Xu Z., Zhu W. (2022). Conserved protein targets for developing pan-coronavirus drugs based on sequence and 3D structure similarity analyses. Comput. Biol. Med..

[65] Roe M.K., Junod N.A., Young A.R., Beachboard D.C., Stobart C.C. (2021). Targeting novel structural and functional features of coronavirus protease nsp5 (3CL(pro), M(pro)) in the age of COVID-19. J. Gen. Virol..

[66] Fang R., Jiang Q., Zhou X., Wang C., Guan Y., Tao J., Xi J., Feng J.M., Jiang Z. (2017). MAVS activates TBK1 and IKKε through TRAFs in NEMO dependent and independent manner. PLoS Pathog..

[67] Wang D., Fang L., Shi Y., Zhang H., Gao L., Peng G., Chen H., Li K., Xiao S. (2016). Porcine Epidemic Diarrhea Virus 3C-Like Protease Regulates Its Interferon Antagonism by Cleaving NEMO. J. Virol..

[68] Perlman S., Netland J. (2009). Coronaviruses post-SARS: Update on replication and pathogenesis. Nat. Rev. Microbiol..

[69] Lin H., Li B., Liu M., Zhou H., He K., Fan H. (2020). Nonstructural protein 6 of porcine epidemic diarrhea virus induces autophagy to promote viral replication via the PI3K/Akt/mTOR axis. Vet. Microbiol..

[70] Lin C.M., Saif L.J., Marthaler D., Wang Q. (2016). Evolution, antigenicity and pathogenicity of global porcine epidemic diarrhea virus strains. Virus Res..

[71] Wang J., Kan X., Li X., Sun J., Xu X. (2022). Porcine epidemic diarrhoea virus (PEDV) infection activates AMPK and JNK through TAK1 to induce autophagy and enhance virus replication. Virulence.

[72] Cottam E.M., Maier H.J., Manifava M., Vaux L.C., Chandra-Schoenfelder P., Gerner W., Britton P., Ktistakis N.T., Wileman T. (2011). Coronavirus nsp6 proteins generate autophagosomes from the endoplasmic reticulum via an omegasome intermediate. Autophagy.

[73] Zhu H., Li Z., Bai J., Jiang P., Wang X., Liu X. (2022). A Systemic Study of Subcellular Localization of Porcine Epidemic Diarrhea Virus Proteins. Pathogens.

[74] Li H., Wang X., Gao D., Huang H., Chen L., Chang H., Wang C., Li Y., Zhao J. (2017). Subcellular Localization and Effect on Type Ⅰ Interferon Response of Porcine Epidemic Diarrhea Virus Nsp7. Acta Vet. Zootech. Sin..

[75] Zhang J., Yuan S., Peng Q., Ding Z., Hao W., Peng G., Xiao S., Fang L. (2022). Porcine Epidemic Diarrhea Virus nsp7 Inhibits Interferon-Induced JAK-STAT Signaling through Sequestering the Interaction between KPNA1 and STAT1. J. Virol..

[76] Zhang J., Fang P., Ren J., Xia S., Zhang H., Zhu X., Ding T., Xiao S., Fang L. (2023). Porcine Epidemic Diarrhea Virus nsp7 Inhibits MDA5 Dephosphorylation to Antagonize Type I Interferon Production. Microbiol. Spectr..

[77] Baddock H.T., Sanja B., Yuliana Y., Malitha R., Marcin B., Lonniep S., Abimael C.M., Haitian F., Keown J.R., Walker A.P.J. (2022). NaR. Characterization of the SARS-CoV-2 ExoN (nsp14ExoN–nsp10) complex: Implications for its role in viral genome stability and inhibitor identification. Nucleic Acids Res..

[78] Li S., Wang L., Wen Y., Han J., Hou J., Hou Z., Xie J., Li H., Li X., Yang Y. (2025). Porcine epidemic diarrhea virus nsp14 inhibited IFN-Ⅰ production by targeting RIG-I for degradation. Virology.

[79] Meylan E., Tschopp J., Karin M. (2006). Intracellular pattern recognition receptors in the host response. Nature.

[80] Shi Y., Tong X., Ye G., Xiu R., Peng G.J. (2020). Structural Characterization of the Helicase Nsp10 Encoded by Porcine Reproductive and Respiratory Syndrome Virus. J. Virol..

[81] Bouvet M., Lugari A., Posthuma C.C., Zevenhoven J.C., Morelli X.J. (2014). Coronavirus Nsp10: A Critical Co-Factor for Activation of Multiple Replicative Enzymes. J. Biol. Chem..

[82] Lin S., Chen H., Chen Z., Yang F., Ye F., Zheng Y., Yang J., Lin X., Sun H., Wang L.J. (2021). Crystal structure of SARS-CoV-2 nsp10 bound to nsp14-ExoN domain reveals an exoribonuclease with both structural and functional integrity. Nucleic Acids Res..

[83] Zhu L., Liu S., Zhuo Z., Lin Y., Zhang Y., Wang X., Kong L., Wang T. (2022). Expression and immunogenicity of nsp10 protein of porcine epidemic diarrhea virus. Res. Vet. Sci..

[84] Bouvet M., Imbert I., Subissi L., Gluais L., Canard B., Decroly E. (2012). RNA 3′-end mismatch excision by the severe acute respiratory syndrome coronavirus nonstructural protein nsp10/nsp14 exoribonuclease complex. Proc. Natl. Acad. Sci. USA.

[85] Wu Y., Zhang H., Shi Z., Chen J., Feng L. (2020). Porcine Epidemic Diarrhea Virus nsp15 Antagonizes Interferon Signaling by RNA Degradation of TBK1 and IRF3. Viruses.

[86] Ren W., Liao Y., Ding X., Jiang Y., Yan J., Xia Y., Tan B., Lin Z., Duan J., Jia X. (2019). Slc6a13 deficiency promotes Th17 responses during intestinal bacterial infection. Mucosal Immunol..

[87] Gao B., Gong X., Fang S., Weng W., Liao Y. (2021). Inhibition of anti-viral stress granule formation by coronavirus endoribonuclease nsp15 ensures efficient virus replication. PLOS Pathog..

[88] Menachery V.D., Debbink K., Baric R.S. (2014). Coronavirus non-structural protein 16: Evasion, attenuation, and possible treatments. Virus Res..

[89] Hou Y., Ke H., Kim J., Yoo D., Su Y., Boley P., Chepngeno J., Vlasova A.N., Saif L.J., Wang Q.J. (2019). Engineering a live attenuated pedv vaccine candidate via inactivation of the viral 2-o 1 methyltransferase and the endocytosis signal of the spike protein 2 3 downloaded from. J. Virol..

[90] Shi P., Su Y., Li R., Liang Z., Dong S., Huang J. (2019). PEDV nsp16 negatively regulates innate immunity to promote viral proliferation. Virus Res..

[91] Feder M., Pas J., Wyrwicz L.S., Bujnicki J.M. (2003). Molecular phylogenetics of the RrmJ/fibrillarin superfamily of ribose 2′-O-methyltransferases. Gene.

[92] Hillen H.S., Kokic G., Farnung L., Dienemann C., Tegunov D., Cramer P. (2020). Structure of replicating SARS-CoV-2 polymerase. Nature.

[93] Wang Q., Wu J., Wang H., Gao Y., Liu Q., Mu A., Ji W., Yan L., Zhu Y., Zhu C. (2020). Structural Basis for RNA Replication by the SARS-CoV-2 Polymerase. Cell.

[94] Deming D.J., Graham R.L., Denison M.R., Baric R.S. (2007). Processing of Open Reading Frame 1a Replicase Proteins nsp7 to nsp10 in Murine Hepatitis Virus Strain A59 Replication. J. Virol..

[95] Mahamaya B., Stephen D., Duo X., Nelli K., Jiuwei L., Jian F., Gregor B., Rong H., Jikui S. (2021). Two conserved oligomer interfaces of NSP7 and NSP8 underpin the dynamic assembly of SARS-CoV-2 RdRP. Nucleic Acids Res..

[96] Kaewborisuth C., He Q., Jongkaewwattana A. (2018). The Accessory Protein ORF3 Contributes to Porcine Epidemic Diarrhea Virus Replication by Direct Binding to the Spike Protein. Viruses.

[97] Ye S., Li Z., Chen F., Li W., Guo X., Hu H., He Q. (2015). Porcine epidemic diarrhea virus ORF3 gene prolongs S-phase, facilitates formation of vesicles and promotes the proliferation of attenuated PEDV. Virus Genes.

[98] Zou D., Xu J., Duan X., Xu X., Li P., Cheng L., Zheng L., Li X., Zhang Y., Wang X. (2019). Porcine epidemic diarrhea virus ORF3 protein causes endoplasmic reticulum stress to facilitate autophagy. Vet. Microbiol..

[99] Wu Z., Cheng L., Xu J., Li P., Li X., Zou D., Zhang Y., Wang X., Wu X., Shen Y. (2020). The accessory protein ORF3 of porcine epidemic diarrhea virus inhibits cellular interleukin-6 and interleukin-8 productions by blocking the nuclear factor-κB p65 activation. Vet. Microbiol..

[100] Kaewborisuth C., Koonpaew S., Srisutthisamphan K., Viriyakitkosol R., Jaru-Ampornpan P., Jongkaewwattana A. (2020). PEDV ORF3 Independently Regulates IκB Kinase β-Mediated NF-κB and IFN-β Promoter Activities. Pathogens.

[101] Walter P., Ron D. (2011). The unfolded protein response: From stress pathway to homeostatic regulation. Science.

[102] Gong X., Feng S., Wang J., Gao B., Xue W., Chu H., Fang S., Yuan Y., Cheng Y., Liao M. (2025). Coronavirus endoribonuclease nsp15 suppresses host protein synthesis and evades PKR-eIF2α-mediated translation shutoff to ensure viral protein synthesis. PLoS Pathog..

[103] Yan L., Huang Y., Ge J., Liu Z., Lu P., Huang B., Gao S., Wang J., Tan L., Ye S. (2022). A mechanism for SARS-CoV-2 RNA capping and its inhibition by nucleotide analog inhibitors. Cell.

[104] Jin X., Chen Y., Sun Y., Zeng C., Wang Y., Tao J., Wu A., Yu X., Zhang Z., Tian J. (2013). Characterization of the guanine-N7 methyltransferase activity of coronavirus nsp14 on nucleotide GTP. Virus Res..

[105] Deng X., Hackbart M., Mettelman R.C., O’Brien A., Mielech A.M., Yi G., Kao C.C., Baker S.C. (2017). Coronavirus nonstructural protein 15 mediates evasion of dsRNA sensors and limits apoptosis in macrophages. Proc. Natl. Acad. Sci. USA.

[106] Hackbart M., Deng X., Baker S.C. (2020). Coronavirus endoribonuclease targets viral polyuridine sequences to evade activating host sensors. Proc. Natl. Acad. Sci. USA.

[107] Saramago M., Costa V.G., Souza C.S., Bárria C., Domingues S., Viegas S.C., Lousa D., MSoares C., MArraiano C., Matos R.G. (2022). The nsp15 Nuclease as a Good Target to Combat SARS-CoV-2: Mechanism of Action and Its Inactivation with FDA-Approved Drugs. Microorganisms.

[108] Kindler E., Gil-Cruz C., Spanier J., Li Y., Wilhelm J., Rabouw H.H., Züst R., Hwang M., V’kovski P., Stalder H. (2017). Early endonuclease-mediated evasion of RNA sensing ensures efficient coronavirus replication. PLoS Pathog..

[109] Chen Y., Su C., Ke M., Jin X., Xu L., Zhang Z., Wu A., Sun Y., Yang Z., Tien P. (2011). Biochemical and structural insights into the mechanisms of SARS coronavirus RNA ribose 2′-O-methylation by nsp16/nsp10 protein complex. PLoS Pathog..

[110] Züst R., Cervantes-Barragan L., Habjan M., Maier R., Neuman B.W., Ziebuhr J., Szretter K.J., Baker S.C., Barchet W., Diamond M.S. (2011). Ribose 2′-O-methylation provides a molecular signature for the distinction of self and non-self mRNA dependent on the RNA sensor Mda5. Nat. Immunol..

[111] Lee C., Kim Y., Jeon J. (2016). JNK and p38 mitogen-activated protein kinase pathways contribute to porcine epidemic diarrhea virus infection. Virus Res..

[112] Guo L., Luo X., Li R., Xu Y., Zhang J., Ge J., Bu Z., Feng L., Wang Y. (2016). Porcine Epidemic Diarrhea Virus Infection Inhibits Interferon Signaling by Targeted Degradation of STAT1. J. Virol..

[113] Sun L., Chen H., Ming X., Bo Z., Qian Y. (2021). Porcine Epidemic Diarrhea Virus Infection Induces Caspase-8-Mediated G3BP1 Cleavage and Subverts Stress Granules To Promote Viral Replication. J. Virol..

[114] Huang H., Li Y., Wang L., Song Y., Zhang G. (2022). Membrane proteomic analysis identifies the polarity protein PARD3 as a novel antiviral protein against PEDV infection. J. Proteom..

[115] Sun M., Yu Z., Ma J., Pan Z., Lu C., Yao H. (2017). Down-regulating heat shock protein 27 is involved in porcine epidemic diarrhea virus escaping from host antiviral mechanism. Vet. Microbiol..

